# Supplementary material for: Quantitative phase imaging verification in large field-of-view lensless holographic microscopy via two-photon 3D printing
Source: Sci Rep. 2024 Oct 9;14:23611. doi: 10.1038/s41598-024-74866-8 (PMC11464779; doi:10.1038/s41598-024-74866-8)
Supplement: Supplementary file 1 — Supplementary Material 1 [file 41598_2024_74866_MOESM1_ESM.docx]

**Supplementary material for:**

**Quantitative phase imaging verification in large field-of-view lensless holographic microscopy via two-photon 3D printing**

Emilia Wdowiak^1^*, Mikołaj Rogalski^1^, Piotr Arcab^1^, Piotr Zdańkowski^1^, Michał Józwik^1^, and Maciej Trusiak^1^**

*^1^Warsaw University of Technology, Institute of Micromechanics and Photonics, 8 Sw. A. Boboli St., 02-525 Warsaw, Poland*

** Corresponding author: emilia.wdowiak.dokt@pw.edu.pl*

*** Quantitative Computational Imaging Lab (qcilab.mchtr.pw.edu.pl) PI: maciej.trusiak@pw.edu.pl*


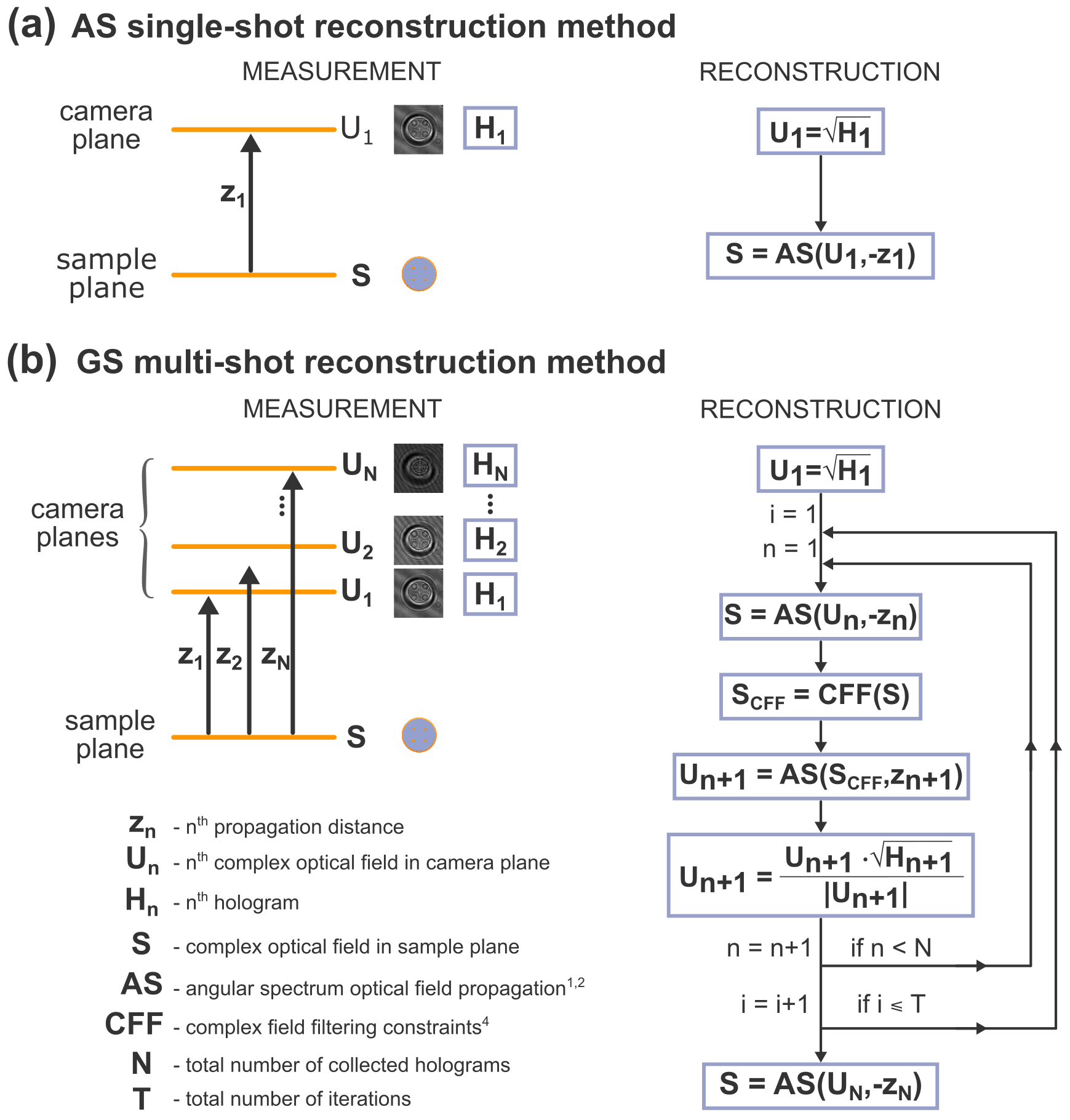


Fig. 1. Flow diagrams of two methods of numerical reconstruction utilized in the study. (a) Angular spectrum (AS) method^1,2^, (b) Gerchberg-Saxton (GS) method^3^ with complex field filtering (CFF) constraints^4^.

Figure 1 compares AS method (a) with GS approach (b). The AS method requires capturing only one hologram at the distance z_1_, that is the smallest possible separation of sample and the camera. Then the complex optical field numerical reconstruction is performed using the AS algorithm^1,2^.

The GS method requires several holograms acquisition with varying sample-camera separation distances (z_1_, z_2_, … z_N_, in our case N = 6). In this scenario the sample remains stationary, and the camera moves away along the optical axis. Reconstruction algorithm starts with H_1_ (the closest hologram). The AS backpropagation is performed to the sample plane by z_1_ distance. Such complex optical field S undergoes the complex field filtering^4^. Resulting complex field S_CFF_ is then forward propagated to the next n+1 hologram plane, providing the new U_n+1_ optical field estimation. Next, the amplitude part of U_n+1_ is replaced with the square root of the n+1 hologram. The abovementioned operations are repeated for each hologram plane what is denoted as single algorithm iteration. The method is performed for user specified number of iterations (T) - in our case 5. Eventually, the final complex optical field is calculated from the last obtained U_N_.

**References**

1. Goodman, J. W. *Introduction to Fourier Optics*. vol. 1 (Introduction to Fourier optics, 3rd ed., by JW Goodman. Englewood, CO: Roberts & Co. Publishers, 2005).

2. Latychevskaia, T. & Fink, H.-W. Practical algorithms for simulation and reconstruction of digital in-line holograms. *Appl. Opt.* **54**, 2424–2434 (2015).

3. Gerchberg, R. A practical algorithm for the determination of plane from image and diffraction pictures. *Optik* **35**, 237–246 (1972).

4. Micó, V., Rogalski, M., Picazo-Bueno, J. Á. & Trusiak, M. Single-shot wavelength-multiplexed phase microscopy under Gabor regime in a regular microscope embodiment. *Sci. Rep.* **13**, 4257 (2023).
