# Supplementary figures and images for: Quantitative phase imaging verification in large field-of-view lensless holographic microscopy via two-photon 3D printing
Source: Sci Rep. 2024 Oct 9;14:23611. doi: 10.1038/s41598-024-74866-8 (PMC11464779; doi:10.1038/s41598-024-74866-8)

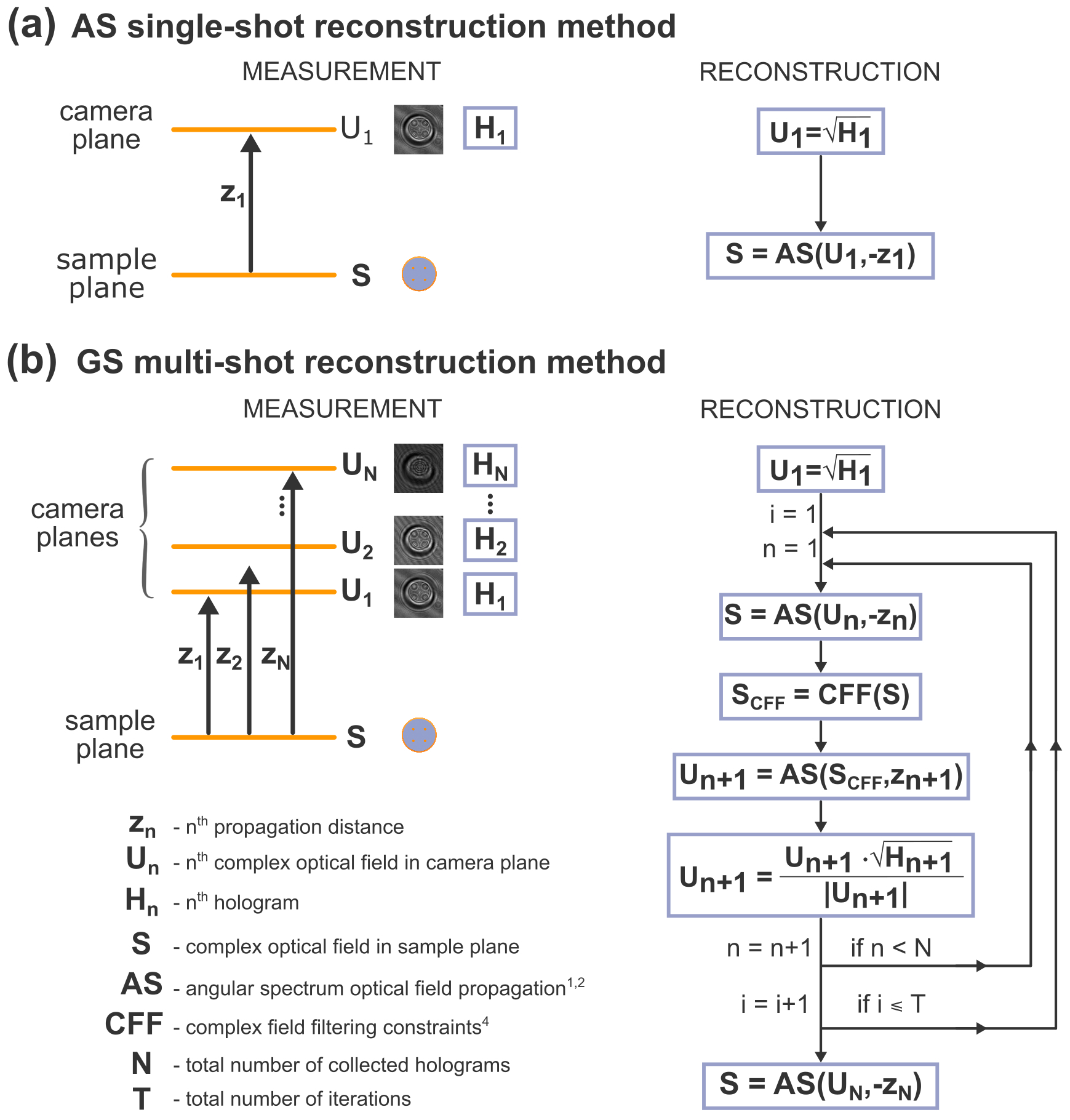

Supplement: Supplementary file 2 — Supplementary Material 2 [file 41598_2024_74866_MOESM2_ESM.jpg]
